# Extensive Genetic Diversity among Clinical Isolates of *Mycobacterium tuberculosis* in Central Province of Iran

**DOI:** 10.1155/2014/195287

**Published:** 2014-11-19

**Authors:** Saman Soleimanpour, Daryoush Hamedi Asl, Keyvan Tadayon, Ali Asghar Farazi, Rouhollah Keshavarz, Kioomars Soleymani, Fereshteh Sadat Seddighinia, Nader Mosavari

**Affiliations:** ^1^PPD Tuberculin Department, Razi Vaccine & Serum Research Institute, Karaj 3197619751, Iran; ^2^Antimicrobial Resistance Research Center, Mashhad University of Medical Sciences, Mashhad, Iran; ^3^Aerobic Bacterial Research and Vaccine Production Department, Razi Vaccine & Serum Research Institute, Karaj, Iran; ^4^Arak University of Medical Sciences, Arak, Iran

## Abstract

Human tuberculosis caused by *Mycobacterium tuberculosis* (*Mtb*) remains a significant disease in many countries. According to Iran's borders with Afghanistan and Pakistan, which are among the 22 high burden countries around the world, this study was conducted to analyze the current molecular epidemiology of tuberculosis and survey genetic diversity of *Mtb* strains in Markazi Province in center of Iran. In this experimental study, 75 sputum specimens and one gastric lavage from all smear-positive TB patients admitted to the public hospitals across the Markazi Province were cultured on specific mycobacterial culture media. Genomic DNA was digested by *Pvu*II and transferred to positively charged nylon membrane by southern blotting method and hybridization by PGRS and DR probes. Genotyping of the isolates by PGRS-RFLP and DR-RFLP displayed a wide range of genetic diversity as 25 and 26 genotypes were identified, respectively. Generally speaking, despite the relatively limited number of isolates in the study, high age of patients and also large heterogeneity found in the setting are both in opposition to active circulation of *Mtb* strains between patients under study either Iranian or Afghan nationals. Thus, it seems that reactivation of latent infection has had the main role in the spread of tuberculosis.

## 1. Introduction

Globally, tuberculosis (TB) remains one of the most prevalent and epidemiologically important diseases of all ages. For three decades since its initial introduction, molecular genotyping has provided the medical community with an enormous load of epidemiological information about TB. This has been materialized through help in identification of index (source) cases in TB outbreaks, detection of TB transmissions, differentiation between cases of relapse and reinfection, and finally effective assessment in antibiotic therapy. According to the World Health Organization report, the prevalence of tuberculosis in Iran in 2012 was estimated to be 33 cases per 100000 people [1]. Geographical spread, racial diversity with large differences in income, and socioeconomic status of people have changed this index in geographic regions and provinces in Iran. In Markazi Province, in 2006, prevalence of TB was estimated to be 10.3 cases per 100000 people which is lower than the national average [2]. Factors such as being neighbors with Tehran Province, located on the main road access to the western areas of the country and a large number of traditional manufacturing and service units that are good purpose of non-Iranian workforce including labor migrants and Afghan asylum seekers, lead to the fact that the province can accommodate a significant number of non-Iranian nationals. On the other hand, political adversity, weak central government, civil wars, terrorism, poverty, and weak health systems for a long time affect neighboring countries of Iran and the presence of nearly 4 million foreign nationals from these countries (about 6 percent of the current population of Iran) has put additional pressure on the structure of Iran health care. As a result, the transmission of infectious diseases like tuberculosis from these countries has changed epidemiological features of these diseases in Iran [3]. Despite numerous studies on molecular epidemiology of tuberculosis in different regions of Iran, there is little information in this regard in Markazi Province in center of Iran. Therefore, the present study was conducted in order to analyze the current epidemiology of TB in this province.

## 2. Materials and Methods

### 2.1. Sampling

75 sputum specimens and one gastric lavage were collected during the period of February 2010 to September 2011 from all smear-positive TB patients (e.g., 72 Iranians and 4 Afghans) admitted to the public hospitals across the Markazi Province, center of Iran.

This study was approved by the Ethics Committee of Arak University of Medical Sciences, Arak, Iran. Study subjects were recruited after providing written informed consent.

### 2.2. Preferential Growth of Bacteria on LJ Medium Containing Glycerol as Compared with Pyruvate LJ Medium

The collected specimens were processed and cultured on glycerinated and also pyruvate traditional Lowenstein-Jensen (LJ) slopes according to the available standard protocols for preliminary identification of* M. bovis* isolates [4, 5].

### 2.3. Genomic Experiments

#### 2.3.1. Genomic DNA Preparations

For IS*6110*-PCR, RD, and PCR-RFLP analyses, simply a loopful of bacterial growth was transferred to a microfuge tube containing 400 *μ*L TB lyses buffer (Cinnagen, Tehran, Iran), heat-treated (95°C, 30 min), vortexed, and centrifuged (4500 g, 15 min) followed by transfer of the supernatant to a new tube and another round of heat treatment (80°C, 30 min). The tube content was then stored at −20°C before use for PCRs. For PGRS-RFLP and DR-RFLP tests, the high quality genomic DNA was extracted as previously described [6].

#### 2.3.2. PCR-16SrRNA

The method of Huard was employed to amplify a 543 bp long fragment of the 16SrRNA. The sequences of primers are shown in Table 1 [7].

#### 2.3.3. PCR-IS6110

The method of McHugh was employed to amplify a 245 bp long fragment of the IS6110 marker using INS1 and INS2 primers (Table 1) [8].

#### 2.3.4. RD-Typing

For the RD experiment, the Warren method was employed with brief modifications where 4 individual PCRs including RD1, RD4, RD9, and finally RD12 were performed by previously described primers to differentiate members of* M. tuberculosis* complex (Table 1) [9].

#### 2.3.5. PGRS-RFLP and DR-RFLP

Internationally standardized protocol was employed to conduct these RFLP experiments [6]. In brief, 6 microgram of genomic DNA was digested by* Pvu*II and incubated at 37°C overnight. DNA fragments were separated by gel electrophoresis and transferred onto a positively charged nylon membrane (southern blot). The digoxigenin tail-labelled PGRS (5′ CGG CCG TTG CCG CCG TTG CCG CCG TTG CCG CCG 3′) or DR (5′ CCG AGA GGG GAC GGA AAC 3′) probes were used for hybridization which was performed at 65°C using rolling bottle method (Table 2). The hybridized membrane was exposed to alkaline phosphatase-conjugated anti-DIG antibody solution and the hybridization signals were detected using substrate BCIP/NBT. Photography was with a scanner (Cannon Laser Base MF3110, Japan) and the acquired image was subsequently saved in JEPG format. The achieved RFLP patterns were carefully observed by two experienced members of the research team and analyzed using Gel-Pro (Media Cybernetics, Milan, Italy) [6, 10].

## 3. Results

### 3.1. Study Population

Out of the 76 specimens (e.g., 76 patients) incorporated in the study, bacterial culture was successfully resulted in collection of 62 mycobacterial isolates. Information of patients including age, gender, and nationality of them were extracted. The patients (culture-positive) include 32 men and 30 women that 4 persons of them were Afghan nationals (2 men and 2 women). Age of the patients varied from 27 to 93 and the average age was 62 years.

### 3.2. Microbial Culture on Glycerinated and Pyruvate LJ

61 isolates had better growth on glycerinated LJ compared with pyruvate LJ that is a sign of* Mycobacterium tuberculosis*. Only one isolate was grown in a pyruvate LJ stronger than glycerinated LJ that showed that it is* Mycobacterium bovis*.

### 3.3. PCR-16SrRNA

All these isolates produced a 543 bp long typical fragment specific to members of Mycobacterium species.

### 3.4. PCR-IS6110 Test

In IS*6110*-PCR all these isolates produced a 245 bp long typical fragment specific to members of* M. tuberculosis* complex.

### 3.5. RD-Typing Test

When the RD typing results were observed, all but one of the isolates in the study setting showed patterns that were matching with* M. tuberculosis* while the non*-M. tuberculosis* isolates were understood to be* M. bovis*.

### 3.6. PGRS and DR RFLP

**Figure 1:**
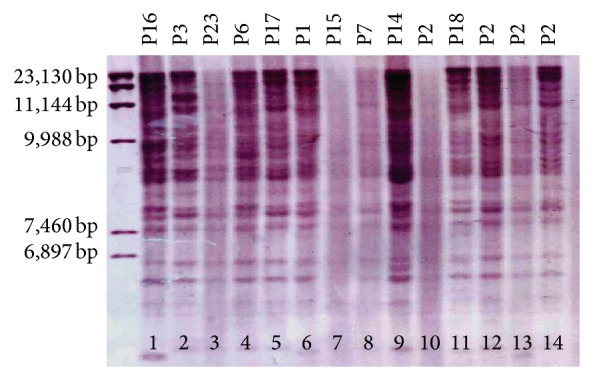
RFLP patterns of* M. tuberculosis* isolates from central province of Iran using* Pvu*II-digested DNA with the PGRS probe. Designations above the lanes represent RFLP-PGRS (P1, 2, 3, 6, 7, 14, 15, 16, 17, 18, and 23) patterns; left side bar is DNA size markers. Lane 1–14:* M. tuberculosis* isolates.

**Figure 2:**
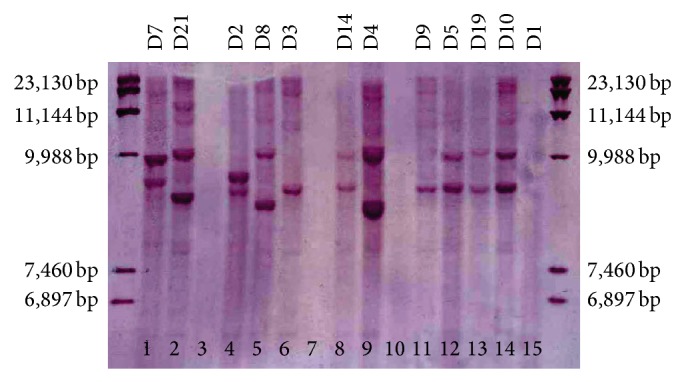
RFLP patterns of* M. tuberculosis* isolates from central province of Iran using* Pvu*II-digested DNA with the DR probe. Designations above the lanes represent DR-PGRS (D1, 2, 3, 4, 5, 7, 8, 9, 10, 14, 19, and 21) patterns; side bars are DNA size markers. Lane 1–14:* M. tuberculosis* isolates.

PGRS-RFLP using* Pvu*II was successfully conducted on 62 isolates and we classified them into 25 genotypic groups (P1 to P25) (Figure 1). This induced eight clustered and 17 orphan patterns. Sixty-two of the isolates were also subjected to RFLP typing using DR probe with* Pvu*II and produced 26 RFLP-DR types (D1 to D26) (Figure 2). This induced eight clustered and 18 orphan patterns. Unlike Iranian patients, none of the Afghan patient isolates had a genetic similarity. Eight isolates belonged to 4 genotypes (2 isolates in per group) in both methods were placed in one group (P5-D1; P4-D6; P1-D11; P8-D20). Genetic patterns of* M. bovis* isolate (P20-D15) were entirely different with* M. tuberculosis* isolates and did not have any similarity with previously reported genotypes from Iran.

## 4. Discussion

Urbanization and industrialization of Central province of Iran caused the migration of many people in this province. Client access to cheap foreign labor, the elimination of insurance costs, and the possibility of further mastery on Afghan workers because of their need to work more than the Iranian jobseekers have caused employers to use them. It leads to the fact that this province can accommodate a significant number of non-Iranian nationals. As a result, the transmission of tuberculosis from these immigrants has changed epidemiological features of this disease in Markazi Province in center of Iran.

In recent decades, molecular epidemiology of tuberculosis in different areas of Iran [11, 12] such as Tehran [13, 14], East Azarbaijan [15], and Khorasan [16] has been studied, but there is little information about Markazi Province. Thus, the present study was conducted in order to analyze the current epidemiology of TB in this province. A considerably large genetic diversity is seen in the population of* Mycobacterium tuberculosis* in Iran confirmed mutually by almost all the recently employed DNA typing systems [15, 17]. Although reactivation of disease due to infection in the past years appears to be explanatory of these epidemiological findings [17], conclusive evidence of this condition is still unknown. Interestingly different epidemiological features of* Mycobacterium bovis* population have been reported in Iran that show very little genetic diversity of this pathogen [18]. The findings of the present study are important from the four aspects. First, viewing 25 and 26 different genetic types by PGRS and DR-RFLP among a relatively small set of* Mycobacterium tuberculosis* isolates (62 isolates) showed considerable variation of this pathogen in Markazi Province. It is essential to note probably increase the number of isolates in further investigation show more polymorphism than the current value. Despite differences in the mechanisms of evolution and changes in PGRS and DR genetic markers, agreement between the results obtained by both typing methods confirms the accuracy of the present results which show a significant level of strain diversity in the Markazi Province. When findings of two genetic markers were merged, four combinational genotypes, namely, P5-D1, P1-D11, P4-D6, and P8-D20, were identified where each type was displayed by two isolates, an indication of their likely epidemiological link. On the other hand, these small cluster patterns indicate that there is reactivation cases TB rather than an epidemic transmission. Second, genetic dissimilarity between* Mycobacterium tuberculosis* strains isolated from Iranian (58 persons) and Afghan (4 persons) patients shows that contrary to popular perception, non-Persian minorities do not have an extensive role in infecting the citizens of Iran. It already has been considered by some authors [3, 17]. Nevertheless, a definite similarity between one Afghan isolate (from a 60-year-old woman) and an Iranian isolate (from a 75-year-old man) shows a single genotype (D11 and P1) therefore likely to be indicative of disease transmission and epidemic isolates. Since a thorough review of the social relationship between the two patients has been done, it was found that Afghan woman worked as a housekeeper in house of Iranian man. Third, considering the average age of patients in research was older than 62 years we believe this is more likely to be a reactivation case of tuberculosis rather than an epidemic transmission event as contact tracing strategy also confirms the explanation of other authors in Iran. Fourth, the only isolate of* Mycobacterium bovis* that was collected from an Iranian patient showed a completely different genetic pattern (P20-D15) compared to those of other* M. bovis* isolates previously genotyped in Iran by Mosavari and colleagues [10]. Considering the age (75-year-old female) and nationality (Iranian) of the patient hosting this specific isolate and the nature of zoonotic tuberculosis caused by* M. bovis* in humans, possibility of infection at an earlier time due to exposure to a strain that for some unknown reasons is no longer frequent or available in cattle farms appears to be explanatory as the most frequent bovine* M. bovis* strains have been exhaustively studied over the recent years in Iran. Therefore, this is more likely to be a reactivation case of zoonotic TB rather than an epidemic transmission event.

## 5. Conclusion

To conclude, in this region role of* M. bovis* in human tuberculosis is little and genetic diversity of* M. tuberculosis* is high, so it seems that more studies like MIRU-VNTR and IS*6110* are required to provide a reliable biogeographical map of TB in this province and Iran.

## Figures and Tables

**Table 1:** The primers sequences used.

Target genes	Primers	Annealing (°C)	Amplicon size (bp)	References
16S rRNA	ACGGTGGGTACTAGGTGTGGGTTTC	62	543	[7]
	TCTGCGATTACTAGCGACTCCGACTTCA			

IS*6110 *	CCTGCGAGCGTAGGCGTCGG	68	123	[8]
	CTCGTCCAGCGCCGC			

RD1	AAGCGGTTGCCGCCGACCGACC	62	146	[9]
	CTGGCTATATTCCTGGGCCCGG			
	GAGGCGATCTGGCGGTTTGGGG			

RD4	ATGTGCGAGCTGAGCGATG	62	172	[9]
	TGTACTATGCTGACCCATGCG			
	AAAGGAGCACCATCGTCCAC			

RD9	CAAGTTGCCGTTTCGAGCC	62	235	[9]
	CAATGTTTGTTGCGCTGC			
	GCTACCCTCGACCAAGTGTT			

RD12	GGGAGCCCAGCATTTACCTC	62	369	[9]
	GTGTTGCGGGAATTACTCGG			
	AGCAGGAGCGGTTGGATATTC			

**Table 2:** Restriction enzyme and probes sequences used for RFLP.

Genes	Restriction enzyme	Probes	Annealing (°C)	References
PGRS	*Pvu*II	CGGCCGTTGCCGCCGTTGCCGCCGTTGCCGCCG	65	[6]
DR		CCGAGAGGGGACGGAAAC	65	[6]
